# Editorial: Molecular level atomistic and structural insights on biological macromolecules, inhibition, and dynamics studies

**DOI:** 10.3389/fmolb.2024.1362215

**Published:** 2024-03-07

**Authors:** Chandrabose Selvaraj, Emilia Pedone, Jung-Kul Lee, Sanjeev Kumar Singh

**Affiliations:** ^1^ Computational and Structural Research in Drug Discovery Lab (CSRDD), Center for Global Health Research, Saveetha Medical College, Saveetha Institute of Medical and Technical Sciences, Chennai, Tamil Nadu, India; ^2^ Institute of Biostructures and Bioimaging, CNR, Naples, Italy; ^3^ Department of Chemical Engineering, Konkuk University, Seoul, Republic of Korea; ^4^ Computer Aided Drug Design and Molecular Modelling Lab, Department of Bioinformatics, Science Block, Alagappa University, Karaikudi, Tamil Nadu, India

**Keywords:** molecular modeling, molecular dynamics, quantum mechanics, atomic insights, drug targets

## Introduction

Atoms are the fundamental components of matter, and when they come together, they create molecules. These molecules can then join to create intricate biological structures. Having a deep understanding of how molecules behave at the atomic level has had a significant influence on the pharmaceutical, biotechnology, and chemical sectors (De Vivo et al. 2016). In various scientific disciplines such as chemistry, physics, materials science, and biology, it is essential to thoroughly examine and comprehend the behaviour, structure, and interactions of atoms and molecules (Selvaraj et al. 2023). In particular, researchers are uncovering novel enzyme structures using different experimental and computational techniques. These methods provide a detailed understanding of how enzymes function at the atomic level, their mechanisms, their roles in reactions, and how they can be inhibited (Carvalho et al. 2014). The atom-level illustrations primarily emphasize enzyme kinetics, inhibition, and the analysis of mutations and conformational changes using quantum mechanical and molecular dynamics techniques (Liu et al. 2018). By uncovering the atomic details of the macromolecule, we can gain insights that will aid in the identification of new agonists or antagonists. This, in turn, could lead to the development of potential drug candidates for the treatment of different diseases (Yu and MacKerell 2017). In order to develop a new inhibitor that specifically targets a particular protein, it is essential to thoroughly understand how the active site of the target protein interacts with potential inhibitors. The main goal in designing a new inhibitor is to fully comprehend the molecular interactions between the inhibitor and the target, improve these interactions to ensure a strong binding and specificity, and rigorously test the effectiveness and safety of the inhibitor (Li and Kang 2020).

The following articles in this Research Topic align with the theme of offering insights at the molecular level to identify drug candidates that can bind to the desired drug targets. This is achieved through various computational methods such as modeling calculations, Quantum Mechanics, and molecular dynamics, which demonstrate the wide range of calculations and predictions. Cao et al. conducted a study to investigate how dapansutrile works on NLRP3 and other protein targets in gouty arthritis. They used bioinformatics analysis and a computer simulation framework. The analysis at the molecular and atomic level, using techniques like molecular docking and molecular dynamics simulations, showed that dapansutrile may not only directly inhibit NLRP3 to reduce the inflammatory response and pyroptosis, but also hinder the movement and activation of inflammatory cells by regulating IL1B, IL6, IL17A, IL18, MMP3, CXCL8, and TNF. Mudedla et al. have applied Quantum-based machine learning and AI models to generate force field parameters for drug-like small molecules. They have applied density functional theory (DFT) calculation for 31,770 small molecules that covered the chemical space of drug-like molecules. They also developed the neural network model for assigning atom types, phase angles, and periodicities. They found that an AI-generated force field was influential in the fast and accurate generation of partial charges and other force field parameters for small drug-like molecules. Papathoti et al. have used the molecular docking and simulation methods for investigating the bioactive compounds extracted from the *Bacillus sp* that target the protein homologs CDC42 of Colletotrichum gloeosporioides causing anthracnose disease in cassava. Five potent compounds from *B. megaterium* were used to target the protein. The interaction of β-sitosterol and phenylacetic acid with the critical residue of CDC42 demonstrated that ligands may inhibit growth-related functional proteins. They have also constructed the protein-protein interactions network, and from that, they have revealed that targeting the CDC42 protein could impart MAPK (Mitogen-activated protein kinases) signaling pathway. Shaik et al. have come up with a new computational biology dimension to interpret the genotype-protein phenotype relationship between SERPINA1 pathogenic variants with its structural plasticity and functional behaviour with NE ligand molecule contributing to the Alpha-1-antitrypsin deficiency. The molecular docking approach findings have demonstrated that the most missense variants negatively impact the affinity of NE (Neutrophil Elastase) and A1AT binding in a molecular complex, lowering A1AT functionality and contributing to its deficiency. Kamboj et al. have applied Gene expression analysis, molecular docking, and molecular dynamics studies to identify the strong antifungal compounds that show specificity with VelB and THR drug targets to inhibit *Curvularia lunata*. Luštinec et al. have performed the Ab-initio evaluation for evaluating the acid influence on the chemical stability of hydrophilic diglycolamides. Their results show strong theoretical findings on including an acid influence on the diglycolamides chemical structure, treated in the frame of the density functional theory. Spassov et al. have used the molecular dynamics simulation methods for protonated and non-protonated forms of the inhibitors and suggested that the salt bridge has an unexpected role in stabilizing the NMT protein conformation and that this may be a significant factor in mediating its effects on NMT inhibitors potency. Danazumi et al. conducted microsecond-level MD simulations to comprehend the role of quinolinyl oxamide derivative (QOD) and an indole carboxamide derivative (ICD) as antimalarial lead drugs with dual inhibition of falcipain-2 and falcipain-3. Jang et al. have come up with the AI-assisted *de novo* design approach to identify a potent and selective inhibitor for the FLT3/FLT-3 (D835Y) mutant. They have optimized the compound PCW-1001 and generated the 10,416 analogues using the LSTM approach. Achudhan et al. identified the novel nitrilases compounds from a coal metagenome using the *in silico* mining methods. The binding scores produced by the novel nitrilase were approximately similar to those of the other prokaryotic nitrilase crystal structures, with a deviation of ±0.5. Kirubhanand et al. analyzed the bioactive nature of lochnericine against Non-Small Cell Lung Cancer (NSCLC) using various computational approaches such as quantum chemical calculations, molecular docking, and molecular dynamic simulation. Also, they confirmed the molecule’s potential bioactivity based on the band gap energy value associated with bioactive compounds through Frontier Molecular Orbital (FMO). Shaik et al. provide comprehensive computational and structural insights into the genotype-protein phenotype correlation of the PCSK9 (Proprotein convertase subtilisin/kexin type 9) pathogenic variant with a PCSK9 inhibitor monoclonal antibody.

In general, the authors of these articles have used Artificial Intelligence and molecular modeling approaches to bring insightful information on atomistic mechanisms and explore functions of the biological macromolecule using atom-level calculations (Huggins et al. 2012; Selvaraj et al. 2022). Some studies have performed extensive molecular dynamics simulations like microsecond level molecular dynamics simulations and accurate Quantum Mechanical Calculations for understanding the atomic role in molecular mechanisms (Sakkiah et al. 2021). The conclusions of the major articles are based on theoretical approaches from the software and publicly available information, with very little confirmation in laboratory conditions. In the future, the added advantage of experimental findings supporting these theoretical findings is required to confirm these findings.
